# Histone/protein deacetylase 11 targeting promotes Foxp3+ Treg function

**DOI:** 10.1038/s41598-017-09211-3

**Published:** 2017-08-17

**Authors:** Jianbing Huang, Liqing Wang, Satinder Dahiya, Ulf H. Beier, Rongxiang Han, Arabinda Samanta, Joel Bergman, Eduardo M. Sotomayor, Edward Seto, Alan P. Kozikowski, Wayne W. Hancock

**Affiliations:** 1 Department of Cardiothoracic Surgery, Xinhua Hospital, Shanghai Jiao Tong University School of Medicine, Shanghai, 200092 China; 2 Division of Transplant Immunology, Department of Pathology and Laboratory Medicine, and Biesecker Center for Pediatric Liver Diseases, Children’s Hospital of Philadelphia and Perelman School of Medicine, University of Pennsylvania, Philadelphia, PA 19104 USA; 3 Division of Nephrology, Department of Pediatrics, Children’s Hospital of Philadelphia and Perelman School of Medicine, University of Pennsylvania, Philadelphia, PA 19104 USA; 4 Department of Medicinal Chemistry and Pharmacognosy, University of Illinois at Chicago, Chicago, IL 60612 USA; 5 George Washington University Cancer Center, Washington, DC 20037 USA

## Abstract

Current interest in Foxp3+ T-regulatory (Treg) cells as therapeutic targets in transplantation is largely focused on their harvesting pre-transplant, expansion and infusion post-transplantation. An alternate strategy of pharmacologic modulation of Treg function using histone/protein deacetylase inhibitors (HDACi) may allow more titratable and longer-term dosing. However, the effects of broadly acting HDACi vary, such that HDAC isoform-selective targeting is likely required. We report data from mice with constitutive or conditional deletion of HDAC11 within Foxp3+ Treg cells, and their use, along with small molecule HDAC11 inhibitors, in allograft models. Global HDAC11 deletion had no effect on health or development, and compared to WT controls, Foxp3+ Tregs lacking HDAC11 showed increased suppressive function, and increased expression of Foxp3 and TGF-β. Likewise, compared to WT recipients, conditional deletion of HDAC11 within Tregs led to long-term survival of fully MHC-mismatched cardiac allografts, and prevented development of transplant arteriosclerosis in an MHC class II-mismatched allograft model. The translational significance of HDAC11 targeting was shown by the ability of an HDAC11i to promote long-term allograft allografts in fully MHC-disparate strains. These data are powerful stimuli for the further development and testing of HDAC11-selective pharmacologic inhibitors, and may ultimately provide new therapies for transplantation and autoimmune diseases.

## Introduction

Histone/protein deacetylase (HDAC) enzymes developed in metazoans prior to the evolution of histones^1, 2^, consistent with current knowledge that HDACs play important roles in regulating the acetylation and function of many non-histone proteins^3–5^, in addition to regulating chromatin accessibility and nucleosome remodeling by deacetylating lysine residues within histone tails^6^. Studies of the protein acetylome are continuing to advance such knowledge^7, 8^. Our prior work, showing that small molecule pan-HDAC inhibitors (HDACi) can increase Foxp3 acetylation and DNA binding and thereby enhance Foxp3+ T-regulatory (Treg) cell production and suppressive activity^9^, led us to analyze the roles of individual HDACs in Foxp3+ Treg cells. This is a necessary step since pan-HDACi can have undesirable effects that are thought to likely curtail their use beyond oncology^10^. Moreover, while gene deletion and/or pharmacological targeting of some HDACs, e.g. HDAC6^11^ and HDAC9^9, 12^, boosts Treg function, deletion of others, e.g. HDAC3^13^, ablates Treg suppressive function and leads to lethal autoimmunity. In the course of these studies, we have analyzed the biology of HDAC11 in Treg cells.

HDAC11 is the sole member of the class IV family of zinc-dependent HDACs, sharing sequence similarity to both class I and II HDAC proteins, and is the most recently identified and, at 39-kilodaltons, the smallest known HDAC^14^. HDAC11 is highly conserved between species, and is relatively tissue-specific in that high expression is limited to kidney, heart, brain, skeletal muscle, and testis^14^. However, along with HDAC10, HDAC11 is one of the least studied HDAC family members^6^. Recently, HDAC11 was identified as a transcriptional repressor of *Il10* expression in mouse and human antigen-presenting cells (APCs)^15^, and in a separate study, shown to associate with the survival of motor neurons (SMN) complex and regulate mRNA splicing^8^. Another HDAC, HDAC6, can physically associate with HDAC11 in both the cytoplasm and nuceli^14^. The association of HDAC6 and HDAC11 in APCs promotes *Il10* expression^16^. HDAC6 and HDAC11 also interact at the vitamin D receptor to regulate expression of MYC^17^, providing a second example of gene regulation by dynamic complexes that contain both HDAC6 and HDAC11 enzymes. We now report genetic and pharmacologic data as to the role of HDAC11 in Foxp3+ Tregs, and show that HDAC11 targeting can enhance Treg function and induce Treg-dependent suppression of allograft rejection.

## Results

### HDAC11 can co-associate with, and deacetylate, Foxp3

**Figure 1:**
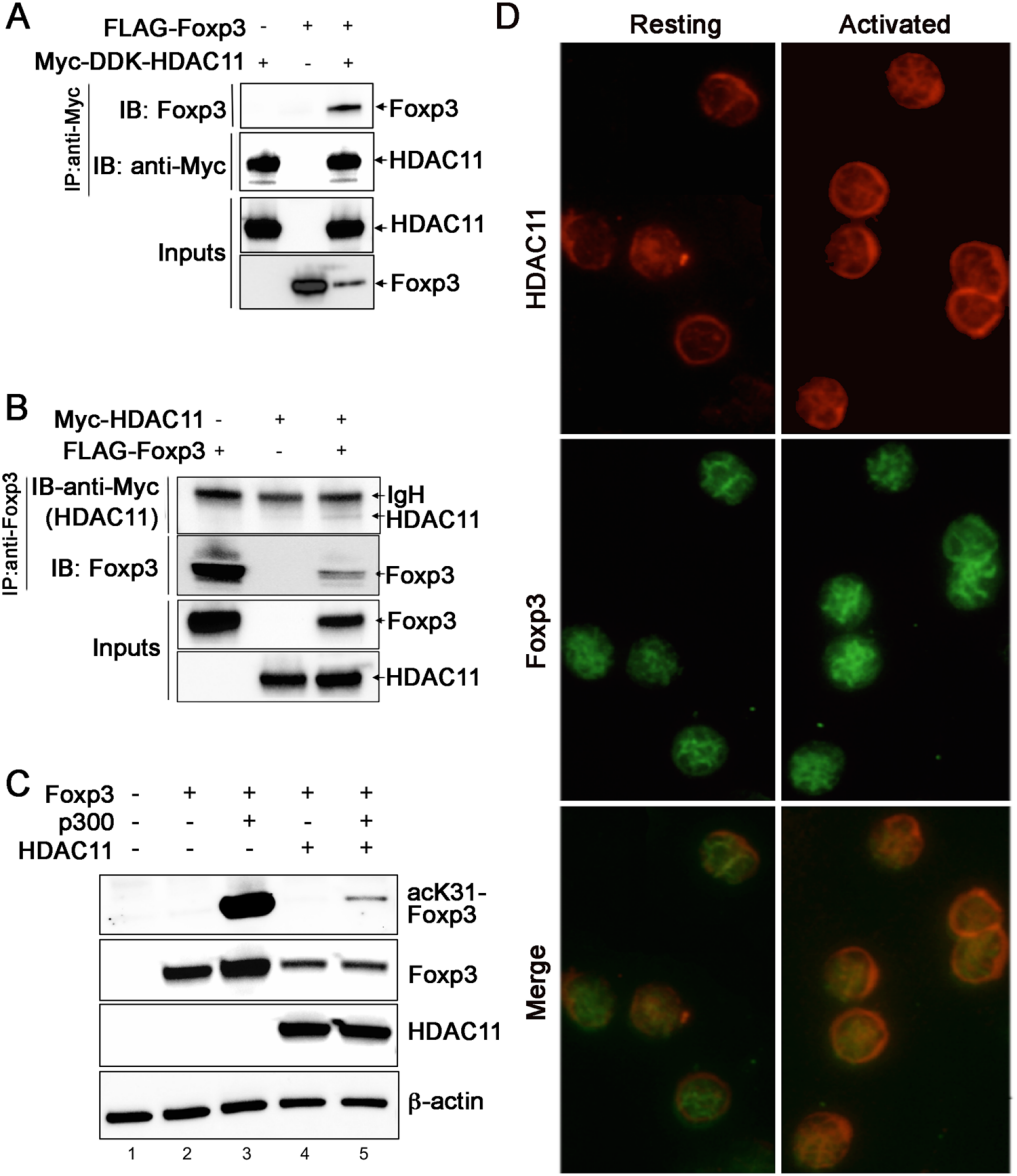
HDAC11 can associate with, and deacetylate, Foxp3. (**A**) Co-immunoprecipitation of Foxp3 protein when HDAC11 is immunoprecipitated from 293 T cells transfected with FLAG-tagged Foxp3 and Myc-tagged Hdac11; original uncropped gels are shown in Supplementary Fig. 1. (**B**) Co-immunoprecipitation of HDAC11 protein when Foxp3 is immunoprecipitated from 293 T cells transfected with FLAG-tagged Foxp3 and Myc-tagged HDAC11; original uncropped gels are shown in Supplementary Fig. 2. (**C**) Western blots showing that the acetylation of lysine-31 (K31) of Foxp3 by p300 (lane 3) is reversed by the presence of HDAC11 (lane 5); original uncropped gels are shown in Supplementary Fig. 3. (**D**) Detection of HDAC11 (nucleus and cytoplasm) and Foxp3 (nucleus) in resting Tregs, whereas CD3/CD28 mAb activation promotes their nuclear co-localization (original magnifications ×400).

Foxp3 plays a central role in controlling Treg development and functions by regulating the expression of several hundred target genes that collectively determine the phenotype and suppressive activity of Treg cells^18^. In addition, the binding of Foxp3 to target genes is regulated by acetylation and deacetylation^9^. These considerations led us to first assess whether Foxp3 could physically interact with HDAC11. After transfection of 293 T cells with Flag-tagged Foxp3 and Myc-tagged HDAC11 constructs, cells were lysed, proteins immunoprecipitated with anti-Myc (HDAC11) antibody, and probed with anti-mouse Foxp3 and anti-Flag antibodies. We found that immunoprecipitation of HDAC11 led to co-precipitation of Foxp3 (Fig. 1A, Supplementary Fig. S1). Comparable results were found when proteins were immunoprecipitated with anti-Flag (Foxp3) antibody, and probed with anti-Myc (HDAC11) antibody (Fig. 1B, (Supplementary Fig. S2). Hence, Foxp3 and HDAC11 can co-associate.

Next, we tested whether HDAC11 could deacetylate Foxp3. Consistent with our previous report^19^, co-transfection of 293 T cells with Foxp3 and p300 led to a marked increase in Foxp3 acetylation (Fig. 1C, lane 3, and Supplementary Fig. S3). Cotransfection of 293 T cells with Foxp3 and HDAC11 led to decreased expression of Foxp3 (Fig. 1C, lane 4), consistent with knowledge that the binding of acetylated Foxp3 to a conserved noncoding sequence (CNS) within a Foxp3 intronic site (CNS2) is required for optimal and stable expression of Foxp3 protein^20^. Importantly, compared to the presence of Foxp3 and p300 (Fig. 1C, lane 3), the addition of HDAC11 decreased both Foxp3 expression and Foxp3 acetylation (Fig. 1C, lane 5). Hence, HDAC11 can bind to Foxp3 and promote its deacetylation.

Lastly, we undertook immunofluorescent localization of HDAC11 and Foxp3 in murine Treg cells. In freshly isolated, unstimulated cells, HDAC11 was detected within both cytoplasmic and nuclear regions, whereas Foxp3 was located almost entirely within the nuclei of Treg cells (Fig. 1D). However, activation with CD3/CD28 monoclonal antibodies (mAbs) led to the nuclear translocation of HDAC11, and co-localization of HDAC11 and Foxp3 within the nuclei of murine Treg cells (Fig. 1D). Hence, *in vitro* studies suggest that HDAC11 might contribute to the regulation of Foxp3 expression and function in Treg cells.

### Deletion of HDAC11 in Foxp3+ Tregs enhances Treg suppressive function

**Figure 2:**
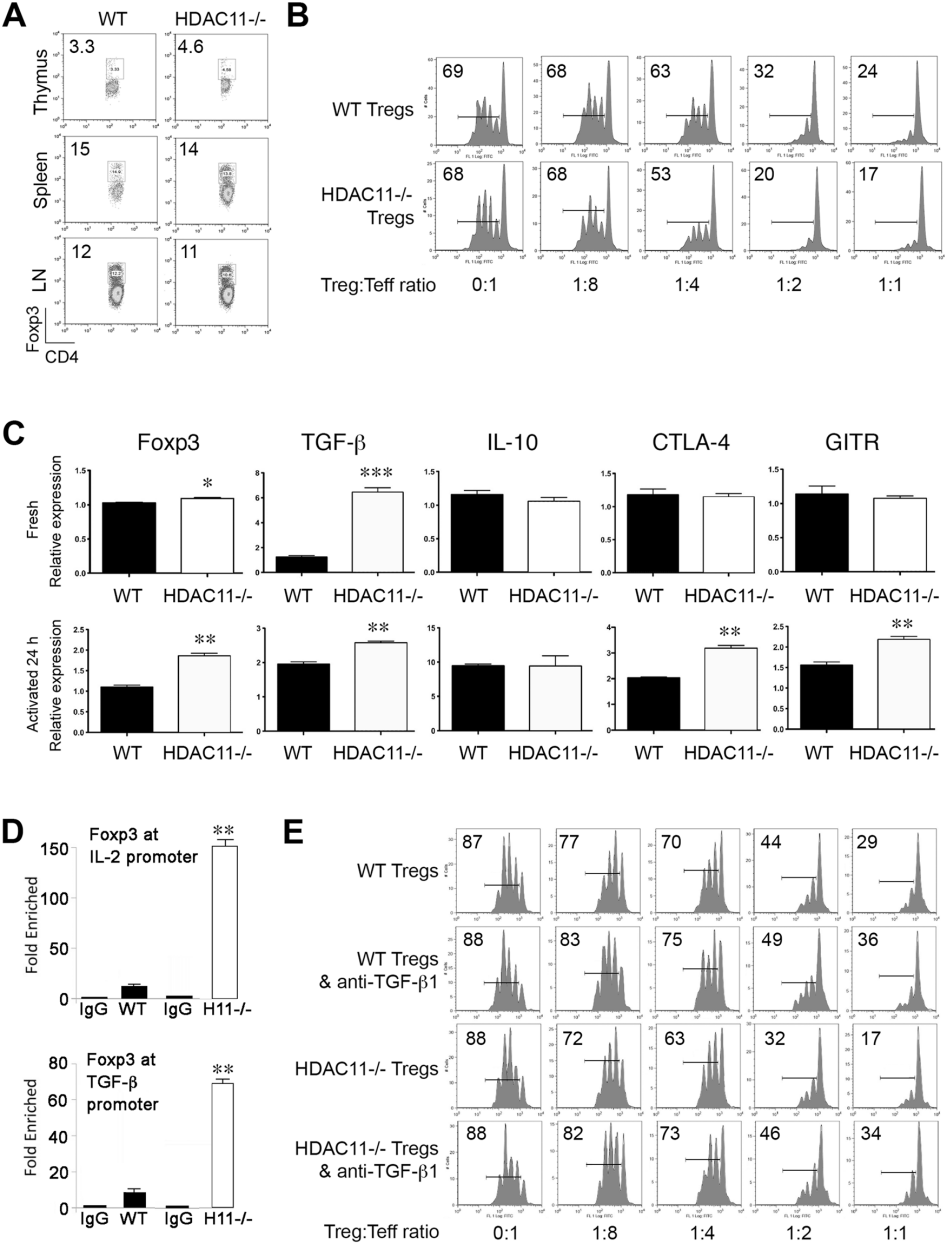
Conditional HDAC11 deletion promotes Foxp3+ Treg suppressive function. (**A**) Deletion of HDAC11 within developing Foxp3+ Tregs did not affect the proportions of thymic, splenic or LN Treg cells; data are from 4 to 6 weeks old male mice, and are representative of results in 4 mice/group. (**B**) *In vitro* Treg suppression assay of the proliferation of CFSE-labeled WT Teff cells using Tregs from WT vs. conditional HDAC11−/− mice, with percentage of proliferating cells shown in each panel, and representative of 4 separate experiments. (**C**) Gene expression (qPCR) by WT vs. conditional HDAC11−/− Tregs at rest or activated with plate-bound CD3/CD28 mAbs for 24 h; normalized gene expression data are shown as mean ± SD, 4 mice/group; *p < 0.05, **p < 0.01, ***p < 0.005. (**D**) ChIP assays comparing levels of Foxp3 binding at the IL-2 and TGF-β promoters in WT and HDAC11−/− Treg cells. (**E**) Enhanced Treg suppressive function *in vitro* is due, at least in part, to increased TGF-β production, as shown by normalization of the suppressive function of conditional HDAC11−/− Treg cells when incubated in the presence of a neutralizing anti-TGF-β mAb; the percentage of proliferating cells is shown in each panel and representative of 3 separate experiments.

We conditionally deleted HDAC11 in Treg cells by crossing *Hdac11* ^fl/fl^ and Foxp3^YFP-Cre^ mice (Supplementary Fig. S4). The resultant (hereafter HDAC11−/−) mice developed normally and had no obvious phenotypic abnormalities. Flow cytometric analysis of T cell populations from the thymus, spleen or lymph nodes showed no significant differences between HDAC11−/− and WT mice (Fig. 2A). However, compared to WT Treg cells, Foxp3+ Treg cells lacking HDAC11 had an increased ability to inhibit the proliferation *in vitro* of conventional CFSE-labeled CD4 + CD25- T-effector (Teff) cells (Fig. 2B).

Analysis of gene expression by real-time quantitative PCR (qPCR) showed that compared to WT Treg cells, freshly isolated HDAC11−/− Tregs had increased expression of Foxp3 (p < 0.05) and TGF-β (b < 0.001) (Fig. 2C). Upon cell activation, in addition to increased Foxp3 and TGF-β expression, HDAC11−/− Tregs showed increased expression of CTLA4 and GITR (p < 0.01), whereas their expression of IL-10 was not significantly different from WT cells under resting or activating conditions (Fig. 2C). ChIP assays showed that the enrichment of Foxp3 at the IL-2 and TGF-β promoters was markedly increased in HDAC11−/− vs. WT Treg cells (Fig. 2D). These data led us to test whether the increased TGF-β production by HDAC11−/− vs. WT Tregs contributed to their greater *in vitro* suppressive function. The addition of a neutralizing anti-TGF-β mAb largely erased the suppressive function of both WT and HDAC11−/− Tregs (Fig. 2E). Hence, HDAC11 deletion enhances expression of multiple Treg-associated genes, including Foxp3, and increases Treg suppressive function *in vitro* through a TGF-β dependent mechanism.

### Deletion of HDAC11 promotes chromatin remodeling and Treg gene expression

**Figure 3:**
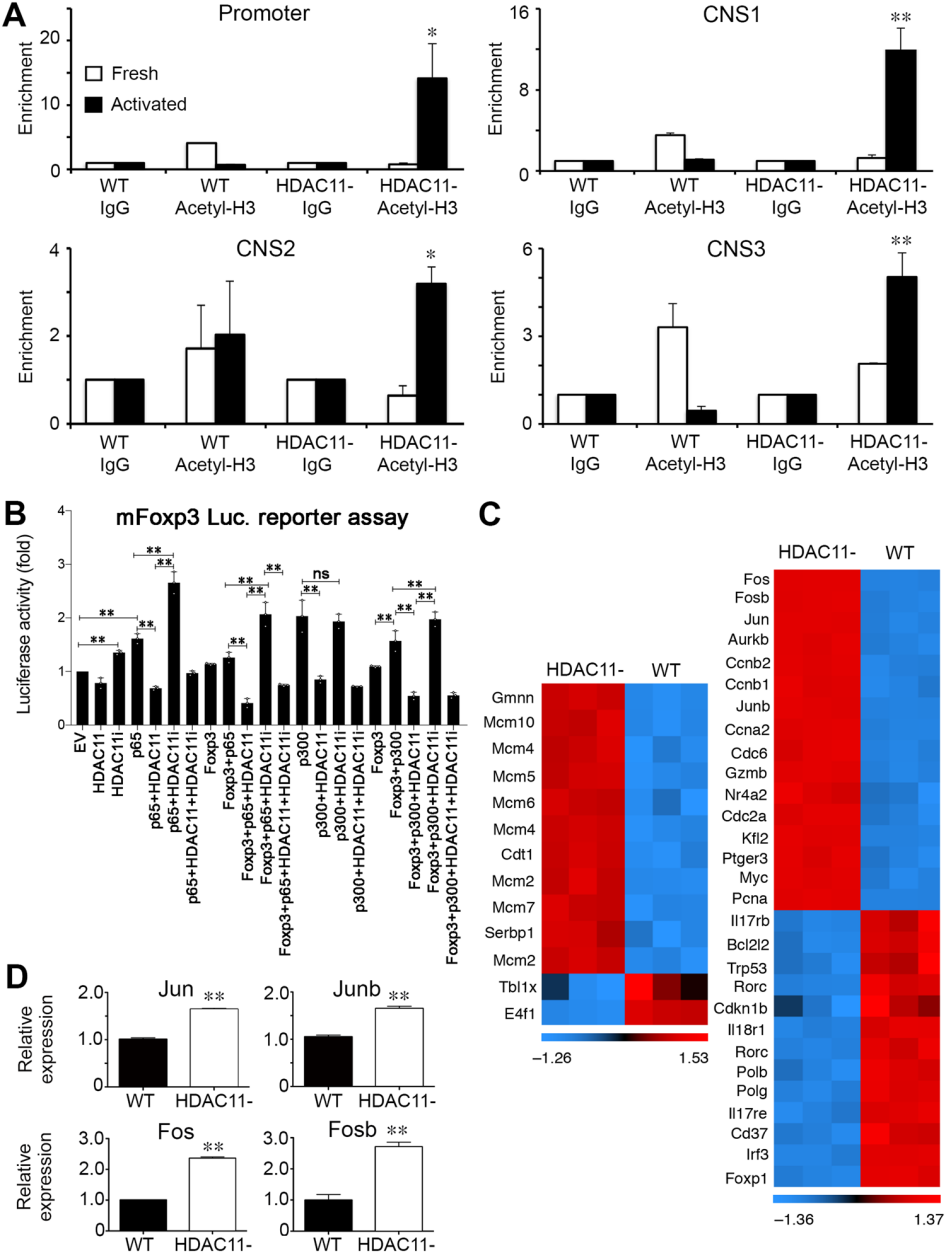
Conditional HDAC11 deletion promotes chromatin remodeling at the Foxp3 locus and Treg gene expression. (**A**) ChIP studies of the Foxp3 promoter and 3 enhancer regions in WT vs. conditional HDAC11−/− Treg cells, showing increased acetyl-H3 binding at all 4 sites in HDAC11−/− vs. WT Treg cells. (**B**) Luciferase reporter assays showing the impact of overexpression, and also inhibition, of HDAC11 in 293 T cells that were transiently transfected with murine Foxp3-promoter-Luc vector plus expression vectors for p65, HDAC11, Foxp3, and p300, as specified, or empty vector (EV) along with 50 ng of pRL-TK Renilla luciferase internal control vector; EV was added to ensure equal amounts of DNA per reaction. After 48 h, cells were treated with PMA (6 ng/ml) and ionomycin (1 μM) for 5 to 6 h, and 1 μM HDAC11i where specified, and cell lysates were harvested for luciferase assays. Firefly luminescence data was normalized to Renilla luminescence and analyzed by ANOVA (*p < 0.05, **p < 0.005); data are representative of the results of 3 or more independent experiments. (**C**) Heat-map of selected genes that were upregulated (red) or downregulated (blue) in conditional HDAC11−/− vs. WT Treg cells. (**D**) qPCR evaluation of WT vs. conditional HDAC11−/− Treg cells, showing increased expression of Jun, Junb, Fos and Fosb in HDAC11−/− Tregs; normalized data are shown as mean ± SD, 4 mice/group, and *p < 0.05, **p < 0.01.

Foxp3 gene expression reflects the contributions of three evolutionarily conserved non-coding sequences (CNS) within Foxp3 intronic regions that function as enhancers, as well as the actions of the Foxp3 promoter itself. ChIP studies assessing acetylated H3-associated chromatin remodeling showed that deletion of Hdac11 increased chromatin accessibility at the Foxp3 promoter and at all 3 Foxp3 enhancer regions (Fig. 3A). Reporter assays using a luciferase-linked Foxp3 promoter showed that HDAC11 decreased the signal driven by NFκB/p65, alone or in the presence of transfected Foxp3, and impaired luciferase activity driven by p65, Foxp3 and the HAT enzyme, p300 (Fig. 3B). The inhibitory effects of HDAC11 at the Foxp3 promoter were reversed by use of JB3-22, an HDAC11 pharmacologic inhibitor (HDAC11i, IC_50_ 0.235 µM) (Fig. 3B). JB3-22 also partially reversed the inhibitory effect of HDAC11 on p300-induced Foxp3 acetylation at lysine 31 (Supplementary Fig. S5), consistent with the ability of HDAC11 to directly regulate acetylation of Foxp3.

We next undertook microarray analyses of HDAC11−/− and WT Tregs to assess HDAC11-dependent global changes in gene expression (Fig. 3C and Supplementary Fig. S6). HDAC11 deletion led to the induction of a series of genes previously linked with DNA replication licensing control, including Cdt1, geminin and at least 6 of the genes associated with the hexameric minichromosome maintenance (MCM) DNA helicase^21, 22^. HDAC11 deletion also decreased expression of additional genes known to interact with, or to be regulated, by HDAC11, including E4f1 and Tblx1^8, 17^. With regard to genes relevant to Treg biology, HDAC11 deletion decreased expression of Rorc and multiple cytokine receptor genes and increased expression of Fos, Fosb, Jun and Junb, whose protein products dimerize to form the AP-1 transcription factor which binds to the Foxp3 promoter^23^, CNS1^24^ and CNS2^25^ regions. Increased expression of Fos, Fosb, Jun and Junb genes in HDAC11−/− vs. WT Tregs was confirmed by qPCR (Fig. 3D). Hence, HDAC11 deletion in Foxp3+ Tregs promotes expression of genes important to protection against oxidative stresses^22^ and important to the development and maintenance of the Treg lineage^23^.

### HDAC11 deletion promotes Treg-dependent allograft survival

**Figure 4:**
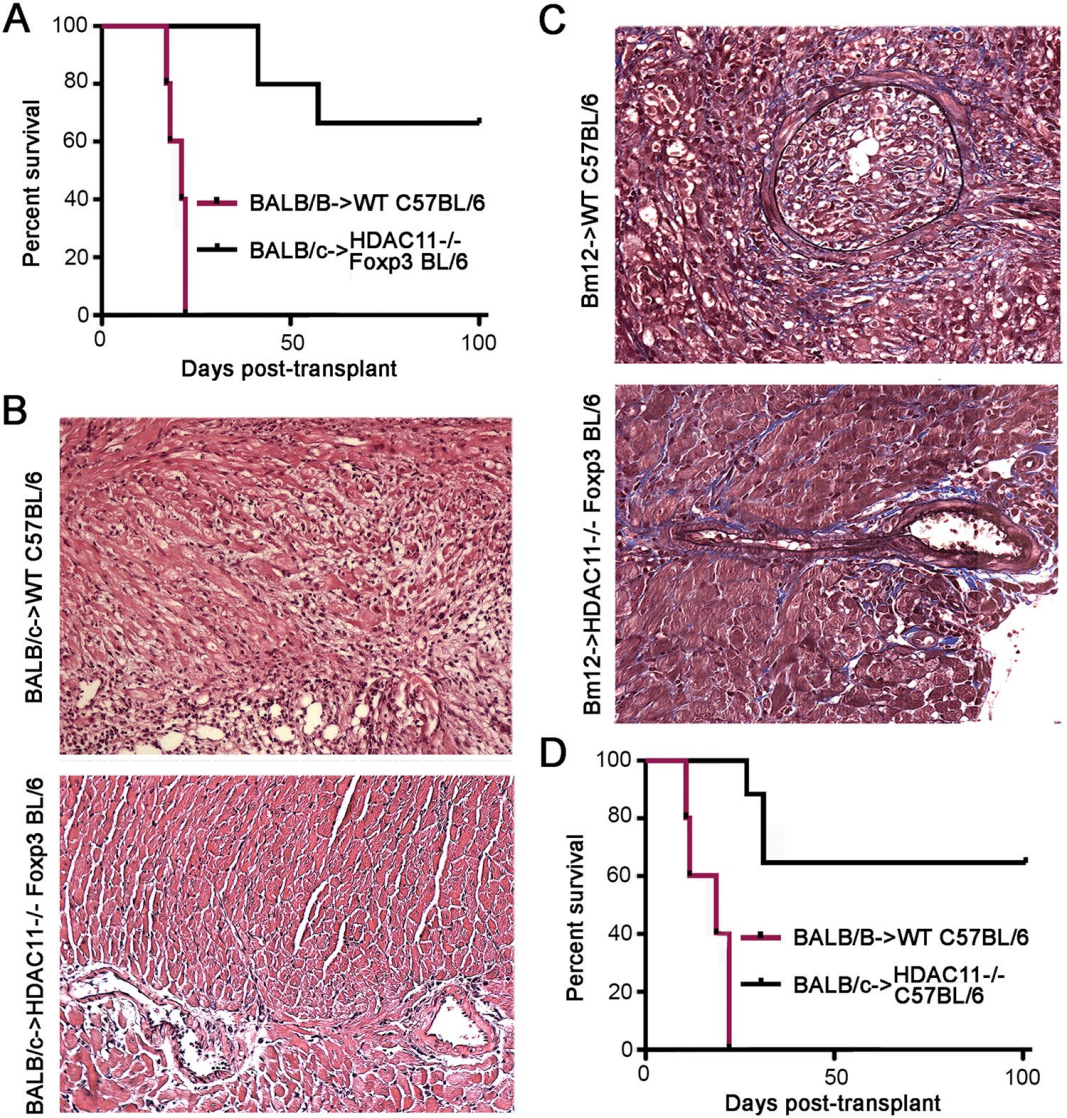
Effects of conditional or global HDAC11 deletion on cardiac allograft survival. (**A**) Compared to the acute rejection of BALB/c cardiac allografts in WT recipients, about two thirds of allografts in mice with conditional deletion of HDAC11 within their Tregs survived for >100 days (p < 0.01, n = 6 allografts/group). (**B**) Histology (H&E) of corresponding cardiac allograft groups as shown in panel (A) when harvested at day 7 post-transplant; note the absence of graft infiltration and myocyte necrosis in HDAC11−/− hosts compared to WT controls. (**C**) Histology of Bm12 cardiac allografts harvested at 30 days post-transplant from WT mice or mice with conditional deletion of HDAC11 within their Foxp3+ Treg cells, with a marked difference in the development of transplant arteriosclerosis (p < 0.001), myocyte injury and mononuclear cell infiltration (see Results for quantitative data on vessel scores for the 2 groups). (**D**) Compared to the acute rejection of BALB/c allografts in WT recipients, about two thirds of cardiac allografts in mice with global deletion of HDAC11 within their Tregs survived for >100 days (p < 0.01, n = 10 allografts/group).

We used 3 murine allograft models to assess the effects of HDAC11 gene deletion *in vivo*. First, we performed cardiac allografting using fully MHC-disparate BALB/c donors (H-2^d^) and C57BL/6 recipients (H-2^b^), comparing survival in WT mice versus those lacking HDAC11 within their Foxp3+ Treg cells. Recipients were treated with a sub-therapeutic regimen of 14 days of rapamycin (RPM, 0.2 mg/kg/d, i.p.), beginning on the day of engraftment, that dampens conventional T cell responses but preserves Foxp3+ Treg cell function^9^. In contrast to cardiac allografts that were acutely rejected in WT mice, mice whose Tregs lacked HDAC11 but were otherwise comparable maintained their grafts long-term (>100 days, p < 0.01) (Fig. 4A). Compared to grafts harvested at day 7 post-transplant from WT recipients that showed diffuse mononuclear cell infiltration and extensive myocytolysis, grafts from mice with HDAC11−/− Tregs showed only small focal mononuclear infiltrates and had well preserved myocardia and vessels (Fig. 4B).

In a second model, WT mice or mice lacking HDAC11 within their Foxp3+ Treg cells were transplanted with class IIa-disparate Bm12 cardiac allografts; recipients received no additional therapy. This class II MHC-mismatched strain combination is widely used to model the development of transplant arteriosclerosis^26, 27^, a major and persistent problem in clinical transplantation^28^. Allografts harvested from WT recipients at 30 d post-transplant showed severe intimal proliferation within muscular arteries (mean vessel score of 2.3 ± 0.3), as well as focal infarcts and dense interstitial mononuclear cell infiltrates, whereas lack of HDAC11 within recipient Tregs led to preservation of myocardial tissues and an absence of intimal proliferation (mean vessel score of 0.2 ± 0.1, p < 001) (Fig. 4C).

In a third model, we tested whether constitutive deletion of HDAC11 affected alloresponsiveness, using the fully MHC disparate BALB/c- > C57BL/6 combination plus 14 d of low-dose RPM therapy (RPM, 0.2 mg/kg/d, i.p.). As with conditional deletion in in Tregs (Fig. 4A), global deletion of HDAC11 protected mice against the acute rejection seen in WT recipients, with 60% surviving long-term (>100 d, p < 0.01) (Fig. 4D). These data show that conditional deletion of HDAC11 within Foxp3+ Tregs can markedly prolong allograft survival across a full MHC disparity, and can protect against the development of transplant arteriosclerosis. The beneficial effects of global deletion in allograft recipients likely have translational significance, given that a small molecule inhibitor of HDAC11 would target conventional T cells and other cellular components of host alloresponses, in addition to the Treg population.

### Pharmacologic targeting of HDAC11 promotes Treg suppression and long-term allograft acceptance

**Figure 5:**
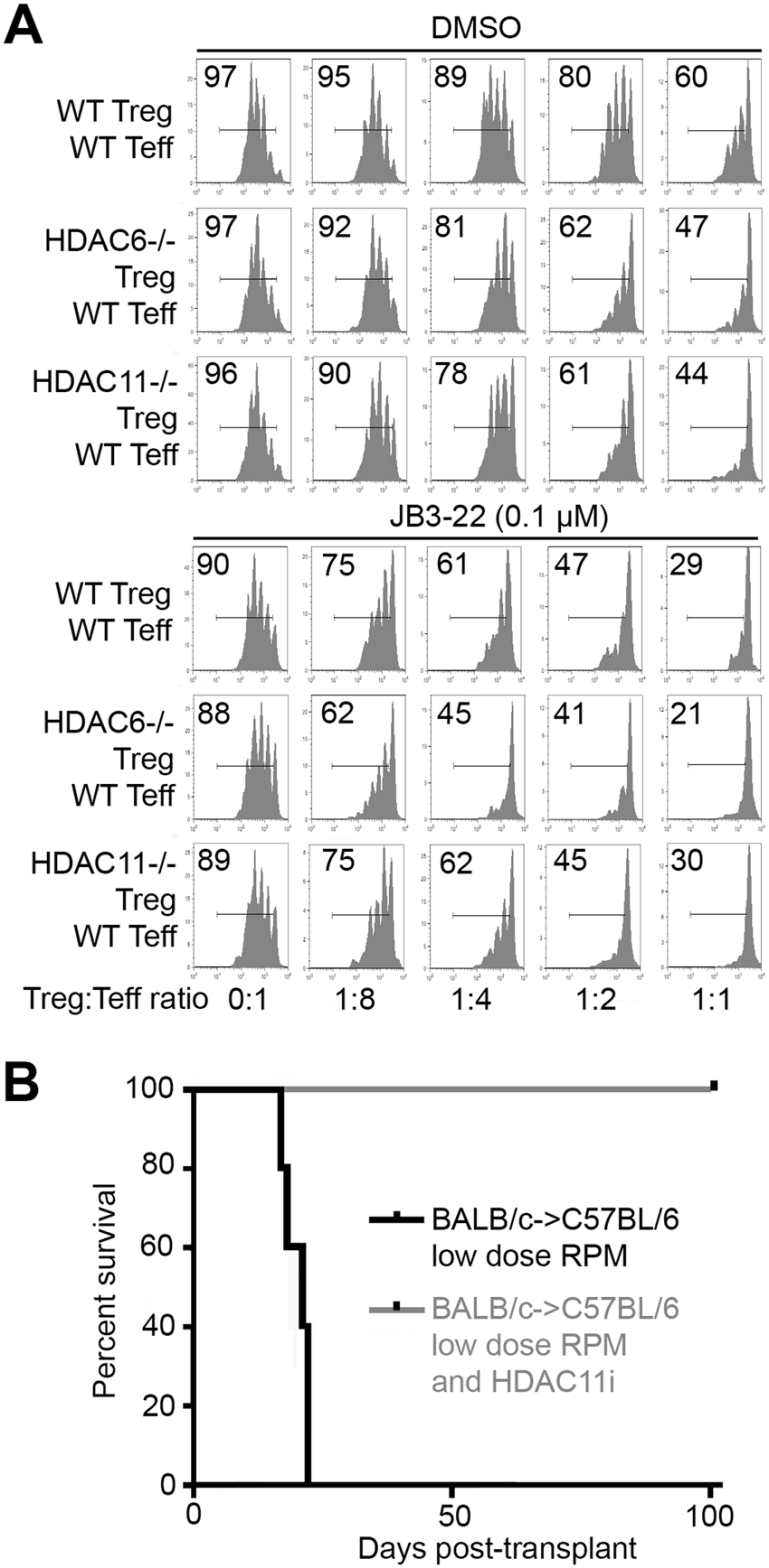
Effects of pharmacologic inhibition of HDAC11 on Treg function and allograft survival. (**A**) Testing of the *in vitro* effects of the HDAC11i, JB3-22, on WT, Hdac6−/− and HDAC11−/− Treg suppressive function. Upper 3 rows show baseline function of WT vs. HDAC6−/− and HDAC11−/− Tregs in the presence of DMSO carrier alone; proliferation of CFSE-labeled conventional T cells is shown in each panel. The lower 3 rows show the effects of HDAC11i (0.1 µM) on Treg function using the same cell types as tested above. The point is that HDAC11i further increases the suppressive function of WT and HDAC6−/− Tregs, but does not have any additional effects on the suppressive function of HDAC11−/− Treg cells. Data are representative of 3 separate assays. (**B**) Effects of HDAC11i (JB3-22, (10 mg/kg/d, 14 d, i.p.) plus low-dose RPM (0.2 mg/kg/d, 14 d) in the BALB/c- > C57BL/6 cardiac allograft model. Control mice receiving low dose RPM rejected their allografts within 2–3 weeks, but cardiac allografts were maintained long-term (>100 days, p < 0.01) in recipients treated for 2 weeks with low dose RPM plus HDAC11i therapy; 6 allografts/group.

The effects of HDAC11 gene deletion of allograft survival (Fig. 4) led us to assess the effects of an HDAC11i (JB3-22) on Treg function *in vitro*, and allograft survival *in vivo*. Our studies were informed by our own (Supplementary Fig. S7) and previous findings that HDAC11 can physically associate, in both the cytoplasm and nucleus, with HDAC6^14^, and that pharmacologic inhibitors of HDAC6 boost Treg function and, in conjunction with a 14-day course of low-dose RPM, promote long-term cardiac allograft survival in murine models (BALB/c- > C57BL/6)^11^. Accordingly, we began by comparing the effects of HDAC11i on WT, HDAC6−/− and HDAC11−/− Tregs *in vitro* (Fig. 5A). Both HDAC6−/− and HDAC11−/− Tregs had increased suppressive function as compared to WT Treg cells, despite addition of DMSO-containing compound diluent, whereas in the absence of Tregs (0:1 ratio) B6 conventional T cells proliferated to a comparable extent (Fig. 5A, upper panel). Addition of the HDAC11i (0.1 µM) significantly increased Treg suppressive function of WT Tregs compared to DMSO-treated Treg cells, and also increased the suppressive function of HDAC6−/− Tregs compared to DMSO-treated Tregs, but had no added benefit when HDAC11−/− Tregs were used (Fig. 5A, lower panel). These data indicate that an HDAC11i was able to increase Treg function in HDAC6−/− Tregs but, consistent with specificity of the compound for HDAC11, had no effect using HDAC11−/− Treg cells. We then tested the HDAC11i (10 mg/kg/d, 14 d, i.p.) plus low-dose RPM (0.2 mg/kg/d, 14 d) in the BALB/c- > C57BL/6 cardiac allograft model. Allografts in WT recipients were acutely rejected, whereas allograft recipients receiving HDAC11i therapy showed long-term (>100 d) cardiac allograft survival (p < 0.01) (Fig. 5B). We conclude that HDAC11 targeting for 2 weeks post-transplant can promote Treg suppressive activity and lead to long-term allograft survival.

## Discussion

Since its discovery, cloning and report of its limited tissue distribution in 2002, HDAC11 garnered relatively little attention compared to most other HDACs and its functions were largely unknown, until two reports in 2009. First, HDAC11 was shown to contribute to control of DNA replication involving sequential recruitment of Cdc6, Cdt1 and several Mcm proteins^29^. HDAC11 deacetylates Cdt1, regulating its ubiquitination and proteasomal degradation, and thereby limits DNA replication. Second, HDAC11 was shown to regulate chromatin accessibility within the *Il10* promoter region, such that knockdown of HDAC11 enhanced antigen-presenting cells (APC) production of IL-10 in response to LPS stimulation^15^. In a follow-up study by the same group, HDAC11 was shown to inhibit the development of myeloid derived suppressor cells (MDSC)^30^.

Moving beyond the role of HDAC11 in myelopoiesis, the current study assessed the role of HDAC11 in Foxp3+ Treg cells. This work was undertaken as part of our ongoing studies of the roles of individual HDAC isoforms in Treg cells, and whether such isoforms may be usefully targeted for therapeutic effect. Deletion of certain isoforms such as HDAC6^11, 31^ and HDAC9^9, 11, 12^ increase Treg function *in vitro* and *in vivo*, though only HDAC6 has been targetable pharmacologically^32, 33^. Deletion of other HDAC enzymes, such as HDAC3^13^ and HDAC5^34^ impaired Treg function, and in the case of HDAC3 led to lethal autoimmunity. Hence, to date, HDAC6 targeting appears to be the best option for pharmacologic enhancement of Treg function, with potential utility in the management of patients undergoing organ transplantation or in patients suffering from autoimmune diseases. The current study presents data showing that genetic and pharmacologic targeting of HDAC11, like that of HDAC6, has therapeutic potential.

Deletion of HDAC11 promotes Foxp3 protein acetylation, which increases its DNA binding^9, 35^. However, additional mechanisms likely also contribute to the increased Treg suppressive function in HDAC11-targeted mice. These include promoting an open chromatin structure at the Foxp3 promoter and all 3 CNS regions within Foxp3 intronic regions; facilitating the actions of transcription factors such as NFκB and AP-1 that regulate Foxp3 expression; and promoting expression of Foxp3 and several other genes important to Treg function, including TGF-β, CTLA-4 and GITR, though not IL-10. The net effect of these effects of HDAC11 deletion was an increase in Treg suppressive function, that *in vitro*, was largely attributable to increased TGF-β production. Treatment of WT Tregs with an HDAC11 inhibitor also increased Treg suppressive function.

Translation of these findings to *in vivo* models was assessed using transplant studies. Remarkably, in conjunction with a 2-week sub-therapeutic course of RPM, deletion of HDAC11, or concomitant use of an HDAC11i in WT recipients, resulted in long-term cardiac allograft survival, as well as protection against the development of transplant arteriosclerosis, a hallmark of chronic allograft dysfunction and a common complication of clinical organ transplantation^28^. Adoptive transfer of human Tregs into chimeric humanized mice was previously shown to protect arterial grafts from developing transplant arteriosclerosis^36^, especially when combined with RPM therapy^37^. The current studies show that comparable protection in a more biologically relevant model can be achieved by pharmacologic therapy. The pathogenesis of transplant arteriosclerosis is complex and multi-faceted, though roles for T and B cells are implicated, in addition to the well-established involvement of macrophages and smooth muscle cells that undergo activation and infiltration into the expanded intima^38, 39^. While additional research will be required to assess the points at which Foxp3+ Tregs modulate such arterial injury and gradual occlusion, beyond their effects on other T cells, as well as monocytes, macrophages, dendritic cells, natural killer cells, neutrophils and B cells, Foxp3+ Tregs are capable of suppressing various deleterious functions of non-immune cells, including that of endothelial and vascular smooth muscle cells, in disease models^40–42^.

Clinical trials involving the adoptive transfer of large numbers of Foxp3+ Tregs are currently at the Phase I stage in various centers worldwide. While no data are yet publicly available, these studies entail considerable expense and expertise to isolate and expand the requisite Foxp3+ Treg populations, and are envisaged in most cases as a single injection of cells in the early post-transplant period. Treg biology is more complex in humans than in mice, and even their isolation and characterization is not straightforward^43^. Thus, unlike in rodents in which Foxp3 has been engineered to be linked with various fluorescent proteins to allow efficient cell isolation, the surface signatures of human Tregs identifiable by flow cytometry are not diagnostic, and conventional T cells can transiently express FOXP3 upon cell activation. As a result, isolated preparations of CD4 + CD25 + CD127^lo^ Tregs used for clinical cell therapy involve purities as low as 76% FOXP3+ cells^44^, and there are important concerns as to the safety of infusion of such mixed cell populations in transplant recipients. An important confounding variable is these trials is use of concomitant immunosuppression with calcineurin inhibitors that are a mainstay of modern immunosuppression, but which impair Treg function even when used at low concentrations^45^. The current studies suggest that the epigenetic regulation of Foxp3 expression and function using a combination of an HDAC11i and RPM may offer considerable advantages over Treg cell therapy, including ease of use, titratable dosing and the option of intermittent or sustained administration.

Lastly, while HDAC11 is overexpressed in various carcinomas compared to normal tissues, its depletion causes inhibition of tumor cell metabolism and their death, and comparable effects are seen when tumor cells are transfected with catalytically inactivated HDAC11 constructs^46^. However, HDAC11 depletion in normal cells does not affect their metabolism or promote their deatth^46^. These findings, along with the data from the current study, including the good health of globally HDAC11-deficient mice, suggest that HDAC11 targeting would not necessarily increase risk of tumor formation or normal cell injury. Such considerations support our ongoing efforts to develop potent and selective HDAC11i compounds that are suitable for clinical therapy.

## Methods

### Mice

We used male WT BALB/c, WT C57BL/6, and MHC class II variant H2-Ab1^bm12^ (Bm12/B6) mice from The Jackson Laboratory, plus Foxp3^YFP-Cre^ mice^47^, and HDAC11^fl/fl^ and globally HDAC11-deleted mice^15^. The latter 3 strains were backcrossed on the C57BL/6 background at least 8 times, and all mice were used at 6–8 weeks of age unless specified. Animal studies were approved and undertaken in accordance with the regulations and guidelines of the Institutional Animal Care and Use Committee of The Children’s Hospital of Philadelphia.

### Antibodies and flow cytometry

Single cell suspensions were stained with mAbs directed against CD4 (BD Bioscience, Pacific blue, clone RM4-5, #558107), CD8 (PE-Cy7, eBioscience, clone 53-6.7, #25-0081), Foxp3 (PE-Cy5, eBioscience, clone FJK-16s, #15-5773), and CD25 (APC, eBioscience, clone PC61.5, #17-0251), and acquired with a Cyan flow cytometer. We also purchased unconjugated CD3 (clone 145-2C11, #553057) and CD28 (clone 37.51, #553294) mAbs from BD Bioscience. Anti-TGF-β mAb that neutralizes isoforms 1, 2 and 3 was purchased from R&D Systems (clone MAB135).

### Treg suppression assays

For *in vitro* studies, 5 × 10^4^ cell-sorted CD4+ YFP+ Tregs, or bead-isolated CD4+ CD25+ Tregs from CD4cre mice, were added to 96-well plates and serially diluted in medium (RPMI-1640 and 10% FBS, plus pen/strep). Thereafter, equal numbers of CFSE-labeled CD4+ CD25- T cells and γ-irradiated antigen-presenting cells were added, plus CD3 mAb (1 μg/ml), and cultured for 3 days. Thereafter, cells were stained with CD4 mAb (Pacific blue) and CFSE and CD4 positive T cell proliferation was acquired and data analyzed with FlowJo.

### Cardiac transplantation and histopathology

Heterotopic abdominal cardiac transplantation was performed using BALB/c or Bm12/B6 donors and C57BL/6 recipients^38^. Recipients of BALB/c cardiac allografts were treated with RPM (0.2 mg/kg/d, i.p.), for 14 days beginning on the day of engraftment, and grafts were collected for histology at day 7 or day 100 post-transplant. In the case of class II-disparate Bm12- > B6 cardiac allografts (using WT or conditional HDAC11−/− recipients), hearts were collected at day 30 post-transplant and all intramyocardial arteries within elastin-stained paraffin sections were scored for the extent of intimal proliferation as <5% occlusion (0), >5–20% (1), >20–40% (2), >40–60% (3), >60–80% (4), or >80–100% (5)^39^.

### Luciferase assays

293 T cells were maintained (37 °C, 5% CO_2_) in RPMI-1640 plus 10% heat-inactivated fetal bovine serum (FBS), penicillin, and streptomycin. Cells at 80% to 90% confluence were transfected with murine Foxp3 promoter luciferase reporter plus expression vectors for HDAC11, p65, Foxp3, p300, or empty vector utilizing Lipofectamine 2000 according to manufacturer’s recommendations. Combinations of expression vectors were utilized wherever specified and empty vector was co-transfected to ensure equal amount of DNA is used per condition. After 48 h, cells were treated with 10 ng/ml phorbol myristate acetate (PMA) and 1 μM ionomycin (Sigma) for 6 h, and luciferase activities of whole-cell lysates were analyzed with a dual-luciferase reporter assay kit (Promega). HDAC11i (JB-3–22) was added at a concentration of 1 μM for 6 h at 48 h post-transfection.

### ChIP assays

ChIP assays were performed using Pierce Magnetic ChIP kit (Cat# 26157). Briefly, TE or Treg cells were fixed with 1% formaldehyde and fragmented by sonication. Chromatin was immunoprecipitated with antibodies directed against Foxp3 (eBioscience # 14-4774-82), HDAC11 (Invitrogen, #PA5-11249) and anti-acetyl-histone H3 (Millipore#06-599). The resultant DNA was purified and used for real-time PCR using SYBR green master mix (Applied Biosystems) and primers for IL-2 promoter, CNS1, CNS2, and CNS3 primer sets (reported previously) and TGFβ1 promoter (forward: GCTCTGAGCCGCACTCG and reverse: CTCCTCGGCTGCTCCTTT).

### Microarray and real-time qPCR

RNA was isolated using RNeasy kits (Qiagen), and RNA integrity and quantity analyzed by NanoDrop ND-1000 and Nanochip 2100 Bioanalyzer (Agilent Technologies). Microarray experiments were performed using whole-mouse-genome oligoarrays (Mouse430a, Affymetrix), and array data analyzed using MAYDAY 2.12 software^48^. Array data were subjected to robust multiarray average (RMA) normalization and analyzed SAM (significance analysis of microarrays). Only data with a false discovery rate adjusted p-value of p < 0.05 and at least 1.5-fold differential expression were included in the analysis. Data underwent z-score transformation for display. Enrichment scores of the up- or downregulated genes were calculated using DAVID Functional Annotation Clustering^49^. Our microarray data was deposited at the Gene Expression Omnibus database (www.ncbi.nlm.nih.gov/geo) and has the accession number GSE95316. Expression of individual genes was verified by qPCR. RNA was reverse transcribed to cDNA (Applied Biosystems), and qPCR performed using Taqman primer and probe sets; data were normalized to endogenous 18 s rRNA and relative expression determined by the formula 2^−ΔCT^.

### Immunoprecipitation and Western blotting

Proteins were isolated using RIPA solution plus protease inhibitor (Sigma). Pull-down antibody was incubated with pre-cleared samples for 2 h at 4 °C, then overnight with protein G agarose. Western blots were performed using anti-Flag and anti-Foxp3 Abs (Cell Signaling).

### Immunofluorescent microscopy

Cytospins of resting Treg cells or Treg cells activated overnight with CD3/CD28 mAbs were fixed, permeabilized with 0.2% Triton-100, blocked with normal goat serum for 2 h, incubated overnight with Abs to Foxp3 and HDAC11, washed, stained with secondary antibodies, and analyzed with a fluorescent microscope.

### Statistics

Data were analyzed using GraphPad Prism 5.0d software. Data were presented as mean ± SD unless specified otherwise. Measurements between two groups were done with a 2-tailed Student’s t-test if data were normally distributed, or Mann-Whitney U unpaired test when the populations were not normally distributed. Groups of three or more were analyzed by 1-way ANOVA with corresponding Tukey’s multiple comparison test if normally distributed, or the Kruskal-Wallis with Dunn’s multiple comparison test if not. Graft survival was evaluated with Kaplan-Meier followed by Log-rank test. P < 0.05 was considered significant. Graphs show mean ± SD, unless otherwise stated in the figure legend.

### Study approval

Animal studies were approved by the Institutional Animal Care and Use Committee of The Children’s Hospital of Philadelphia (protocol 2013-000561).

## Electronic supplementary material

Supplement
