# Supplementary material for: Histone/protein deacetylase 11 targeting promotes Foxp3+ Treg function
Source: Sci Rep. 2017 Aug 17;7:8626. doi: 10.1038/s41598-017-09211-3 (PMC5561267; doi:10.1038/s41598-017-09211-3)
Supplement: Supplementary file 1 — Supplement [file 41598_2017_9211_MOESM1_ESM.pdf]

Histone/protein deacetylase 11 targeting promotes Foxp3+ Treg function

Jianbing Huang[1], Liqing Wang[1], Satinder Dahiya[1], Ulf H. Beier[2], Rongxiang Han[1], Arabinda Samanta[1], Joel Bergman[3], Eduardo M. Sotomayor[4], Edward Seto[4], Alan P. Kozikowski[3], & Wayne W. Hancock[1]*

Supplementary Files

Supplementary Figure S1:
Original uncropped gels for panels shown in Figure 1A

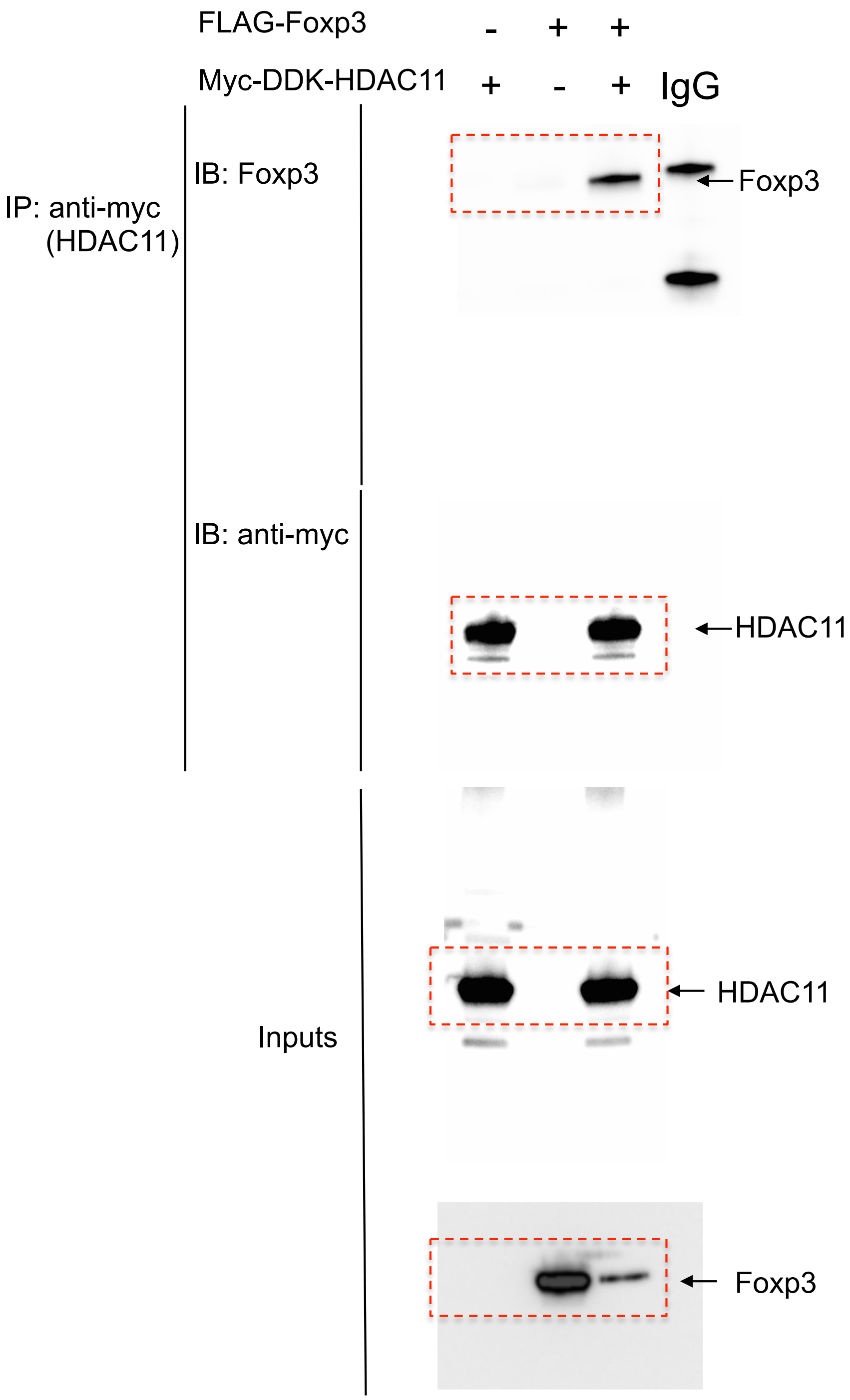

Supplementary Figure S2:
Original uncropped gels for panels shown in Figure 1B

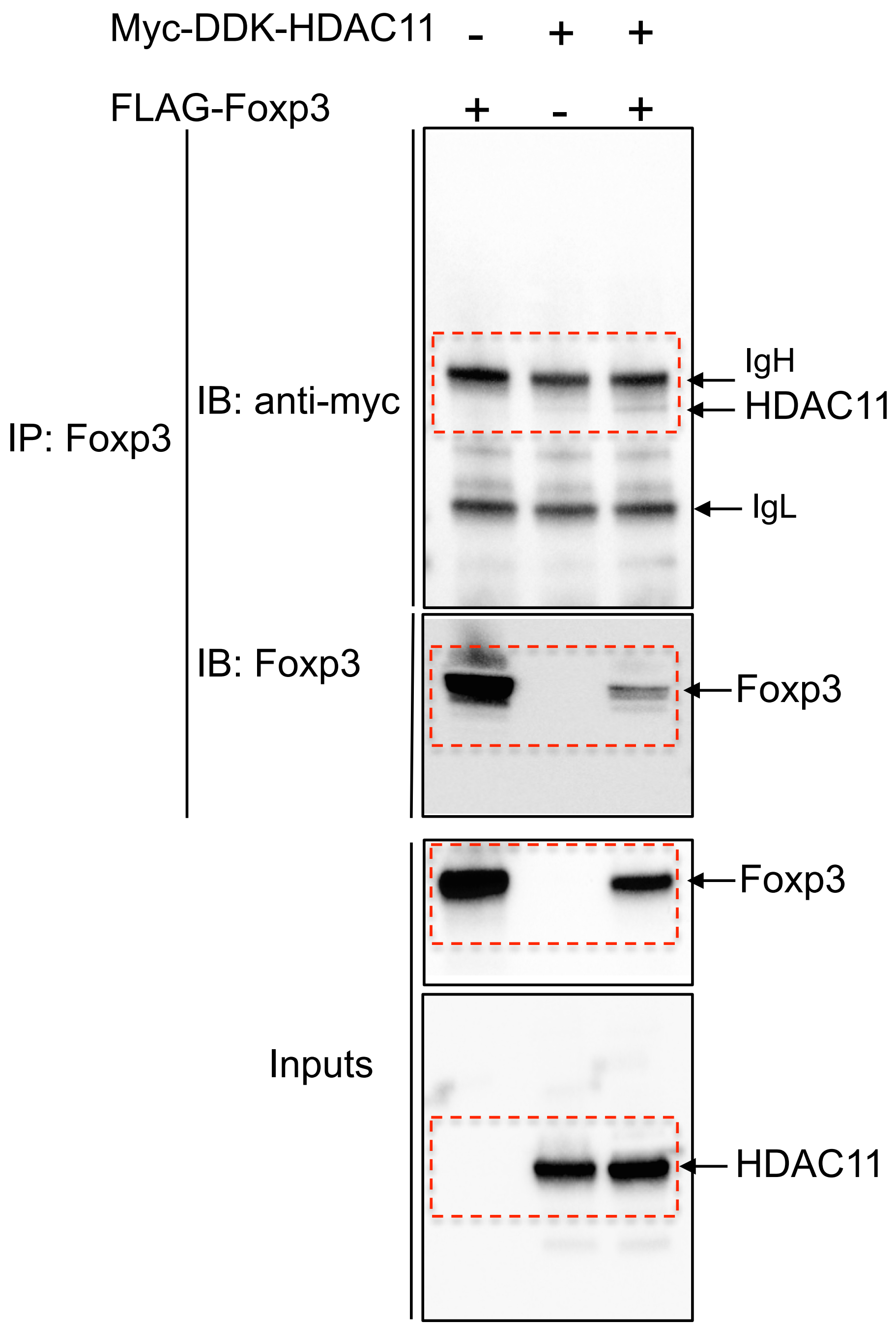

Supplementary Figure S3:
Original uncropped gels for panels shown in Figure 1C

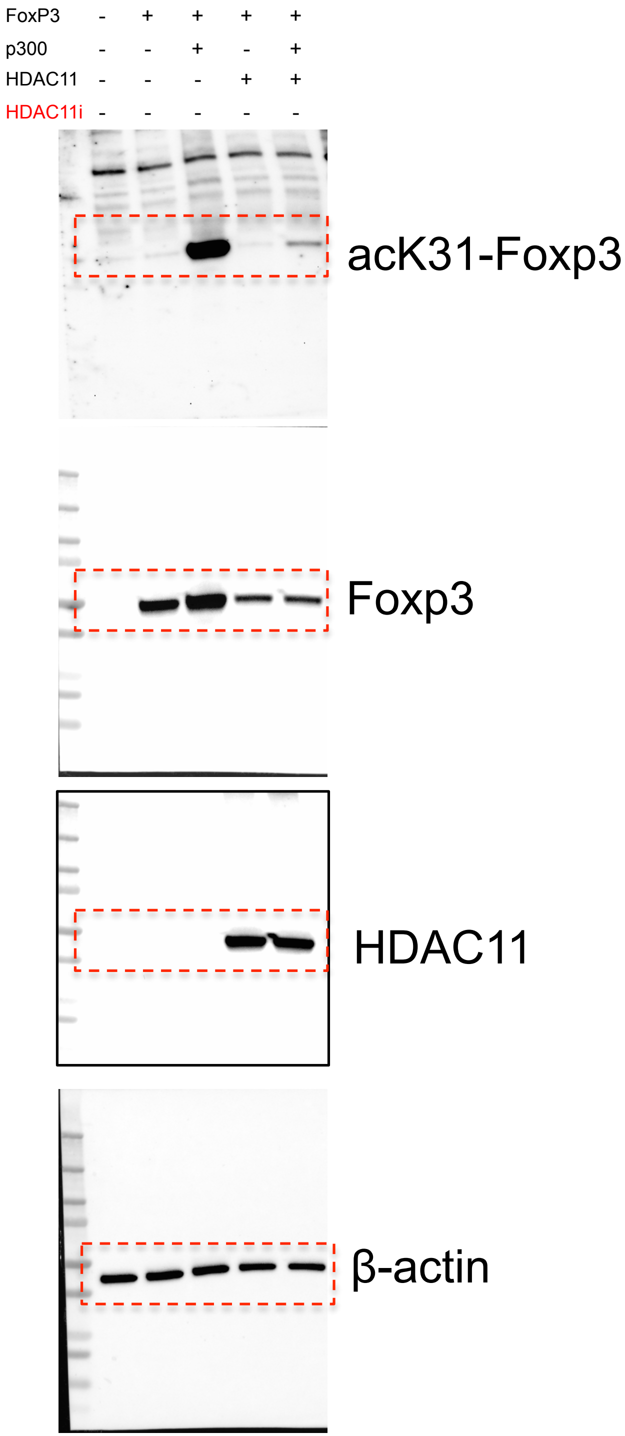

Supplementary Figure S4:
Conditional HDAC11 gene deletion and global HDAC11 gene deletion (qPCR)

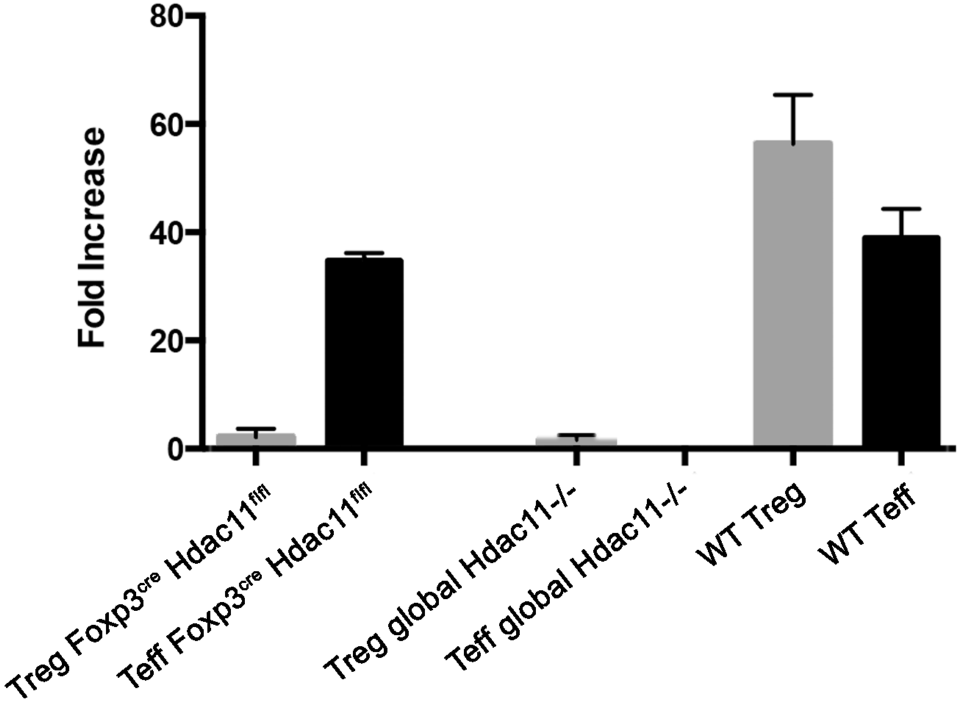

qPCR analysis of HDAC11 gene expression in Treg and Teff cells from WT mice, mice with conditional deletion of HDAC11 in Tregs (Foxp3^{cre}), or global HDAC11 deletion (4 mice/group).

Supplementary Figure S5:
Conditional HDAC11 gene deletion and global HDAC11 gene deletion (qPCR)

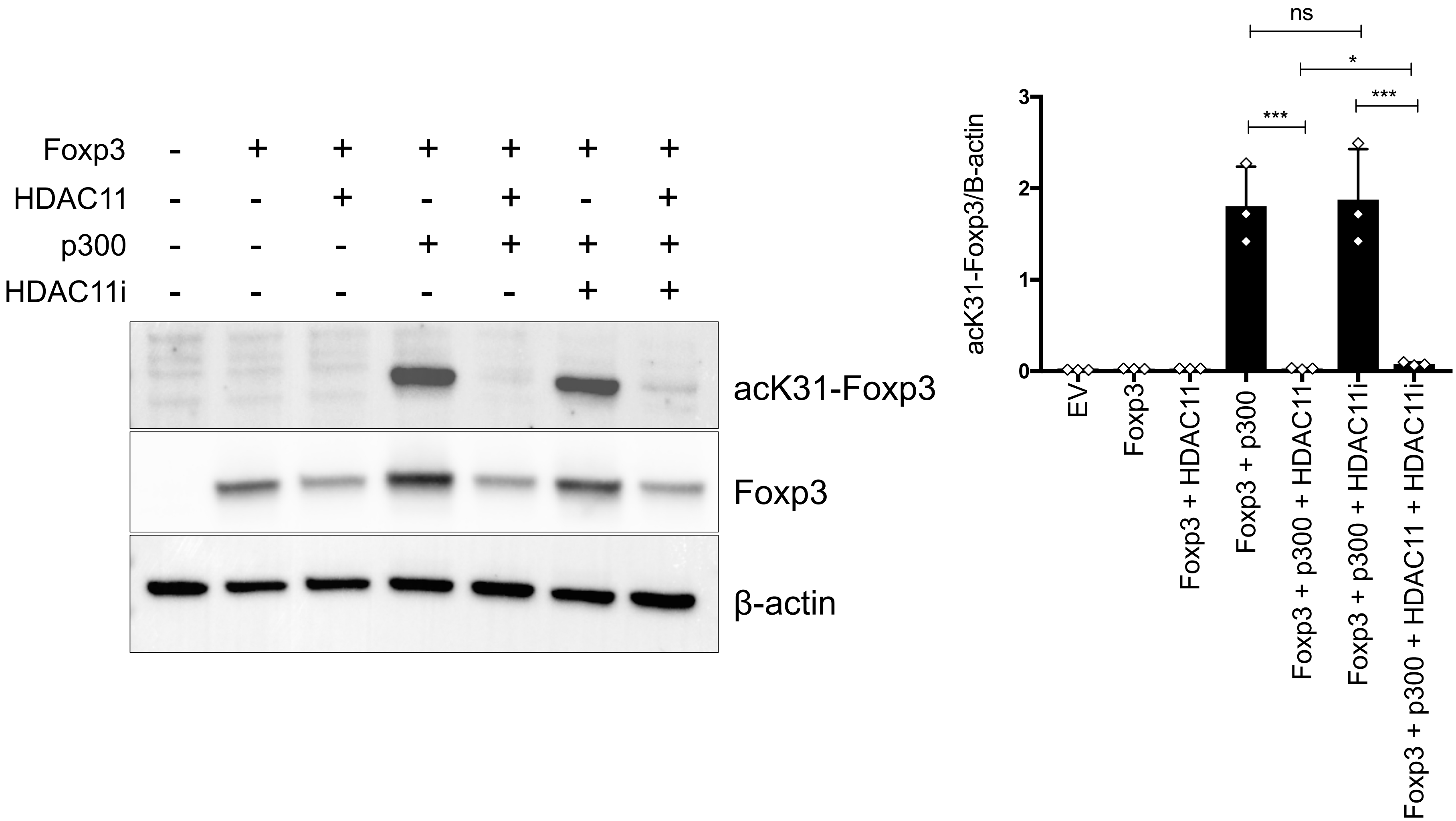

Western blots showing that the ability of p300 to promote acetylation of Foxp3 at lysine-31 (K31) is impaired by HDAC11, whereas use of an HDAC11i partially restored p300-mediated Foxp3 acetylation. The results of densitometric analysis from 3 separate experiments are shown at right.

Supplementary Figure S6:
Functional annotation clustering of the effects of conditional deletion of HDAC11 in Foxp3+ Treg cells

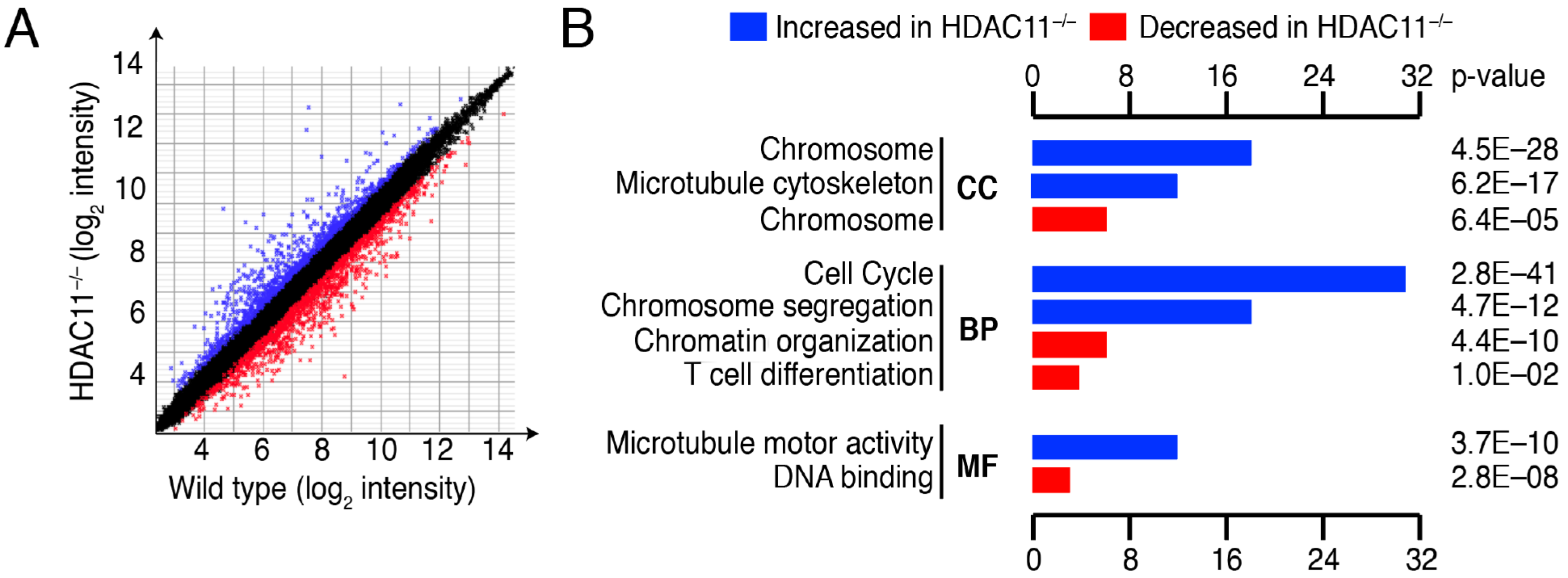

Microarray analysis related to Figure 3C: (A) Scatterplot of log_2 transformed expression matrix data (means from 3/group) from Tregs with or without conditional HDAC11 deletion. Differential expression was determined by SAM with a false discovery rate adjusted p-value of 0.05% and at least 1.5-fold differential expression, with blue and red representing genes up- and downregulated by HDAC11 deletion, respectively. (B) Enrichment scores of the up- or downregulated genes calculated using DAVID Functional Annotation Clustering. CC (cellular component), BP (biological process) and MF (molecular function) indicate gene ontology categories.

Supplementary Figure S7:

Co-association of HDAC6 and HDAC11 in transfected 293T cells

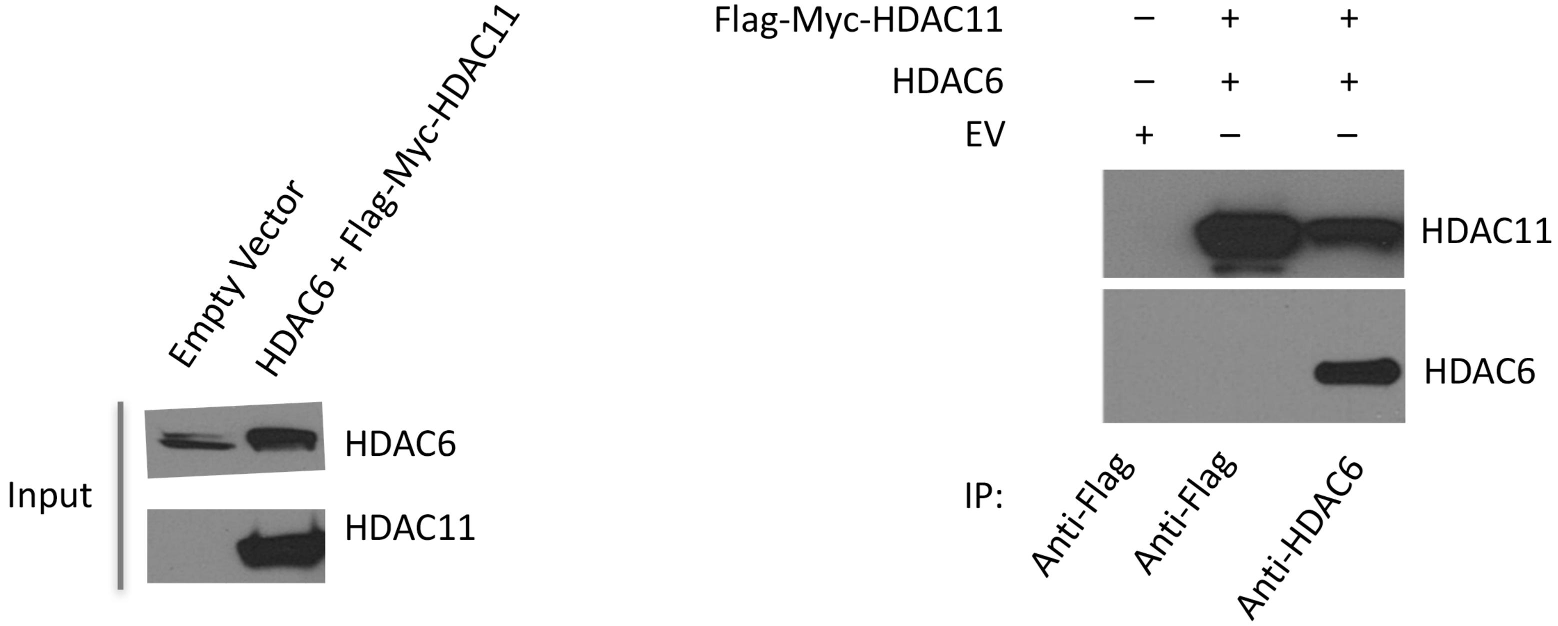

293T cells were transfected with Flag-Myc-HDAC11, followed by immunoprecipitation of HDAC11 using anti-Flag Ab, and Western blot detection of co-precipitated HDAC6.
